# Beyond the record: aligning policy intentions with employment outcomes

**DOI:** 10.3389/fpsyg.2026.1785990

**Published:** 2026-06-01

**Authors:** Cody Normitta Porter, Paul Gavin, Kieran McCartan, Mircea Zloteanu

**Affiliations:** 1 School of Social Sciences, University of the West of England, Bristol, United Kingdom; 2 Department of Psychology, King’s College, London, United Kingdom

**Keywords:** criminal justice, criminal records, employability, employment, policy brief

## Abstract

This policy brief examines the consequences of criminal records, with emphasis on employability research, employment initiatives, and reform recommendations. In the UK, individuals with criminal records—particularly those convicted of violent or sexual offences—remain systematically excluded from the labour market. Employment is one of the strongest predictors of desistance from crime, social stability, and psychological wellbeing following criminal justice involvement. Despite legislative safeguards and well-intentioned initiatives, employment outcomes following release remain persistently low. We argue that reform must move beyond disclosure timing alone and toward structured, evidence-led hiring practices that reflect actual risk, rehabilitation, and proportionality. Without such reform, current approaches risk entrenching stigma, wasting public resources, and undermining reintegration goals.

## 1 Introduction

### 1.1 Employment and recidivism

Employment is consistently identified as a cornerstone of successful reintegration following involvement in the criminal justice system (McGuire, 1999, 2013). Secure and meaningful work is associated with reduced reoffending, improved mental health, and greater social stability. In the United Kingdom ‘UK’, however, individuals with criminal records experience disproportionately high levels of unemployment, driven not only by skills deficits or labour market conditions, but by systemic and psychological barriers embedded in hiring practices (Devins and Foley, 2011; Haslewood-Pócsik et al., 2008).

Despite legislative protections such as the Rehabilitation of Offenders Act 1974 and the availability of criminal record filtering, disclosure remains a major point of exclusion (Unlock, 2024). Employers often lack clarity regarding when, how, and how much information should be requested or considered, resulting in inconsistent practices and risk-averse decision-making. These challenges are intensified for individuals convicted of violent or sexual offences, who face extreme stigma and are often excluded categorically, regardless of offence context, time elapsed, or evidence of rehabilitation.

This policy brief synthesises recent psychological and criminological research to examine how stigma, risk perception, and criminal record disclosure shape employability outcomes. It argues that current policy approaches often prioritise symbolic reassurance over empirical effectiveness and calls for more nuanced, evidence-led reform. The brief concludes with targeted recommendations for policymakers, funders, and researchers.

### 1.2 Criminal record figures

In the UK, holding a criminal record can significantly restrict access to employment, even when offences are minor, historic, or legally spent. The Oswin Project (2024) reported that approximately 1 in 4 working-age people (9.4 to 12.6 million individuals) have a record on the Police National Computer (PNC). In response to a Freedom of Information request by Unlock, the Home Office further confirmed that over 12.5 million individuals had a criminal record recorded on the PNC as of April 2024 (Unlock, 2024). Although overall crime rates fluctuate, these figures indicate that criminal record holding in the UK is both widespread and persistent, creating substantial and enduring barriers to employment, social inclusion, and rehabilitation.

### 1.3 Post-release data

To reduce reoffending and promote reintegration, UK criminal justice policy has increasingly relied on 6-week and 6-month post-release employment statistics as headline indicators of success. However, these metrics capture employment during a period characterised by acute instability, including housing insecurity, license conditions, mental health challenges, and the immediate requirement to disclose a criminal record when seeking work.

The Ministry of Justice (2018) reported that only 17% of individuals released from prison in England and Wales were in employment within six weeks of release. More recent data suggest a modest increase, with employment rates rising from 15% in April 2022 to 19% at the six-week post-release point (Ministry of Justice, 2023). While these figures are often presented as evidence of progress, the consistently low absolute levels of employment – as seen in the raw numbers - indicate that early post-release labour market entry remains structurally difficult. This suggests that criminal record disclosure, employer stigma, and limited access to stable opportunities continue to constrain reintegration during the period when support is most critical.

## 2 Employment statistics

### 2.1 Government data interpretation errors

Percentage-based reporting can be misleading when absolute sample sizes are not considered. For instance, a 10% increase in employment outcomes could mean very different things depending on the size of the cohort. Consider the following example of such an increase for a small or large cohort over a 2-year period, designed to track the effectiveness of employment initiatives.

In a large cohort of 1,000 individuals with criminal records, if 100 of these people gain access to employment in Year 1, and this increases by 10% in Year 2, this equates to 100 more people gaining employment – a successful increase, and one worth funding. However, in a small cohort of 100 individuals, assuming 10 are employed in Year 1, the same 10% employment figure increase in Year 2 reflects only 1 more person gaining employment (i.e., 11 of 100).

Although the percentage change is identical in both scenarios, the absolute impact on the population and on social and economic outcomes is vastly different. Without reporting the raw numbers alongside percentages and percentage change, policymakers may overestimate or underestimate the real-world significance of post-release employment interventions.

Porter et al. (2025c) highlight that reliance on relative percentages alone can exaggerate effectiveness while obscuring persistently low absolute employment rates. They recommend reporting both raw counts and percentages (or ratios) to provide a more accurate representation of post-release employment outcomes.

We also recommend that policymakers, researchers and practitioners integrate absolute figures alongside percentages, enabling policymakers, practitioners, and funders to appropriately evaluate the scale of effect, allocate resources efficiently, and prioritise interventions that yield meaningful, population-level improvements in employment among justice-involved individuals.

### 2.2 Employers misinterpretation and risk perception

Beyond structural barriers, a critical but under-examined issue concerns how post-release employment and reoffending data are interpreted and communicated to employers and policymakers. When employment rates are low (e.g., below 20%), they do little to disrupt underlying stigma or risk-averse hiring practices. Employers who are unsure whether to hire an individual with a criminal record may interpret persistently low employment figures as implicit social information: other employers are not hiring such individuals, as they may possess risk-relevant knowledge that they themselves lack. Such thinking can reinforce defensive decision-making, increasing rejection rates even when empirically assessed reoffending risk is low. These dynamics have important policy implications.

Without careful framing of risk, grounded in psychological theory and empirical evidence, any such approaches may inadvertently strengthen exclusionary attitudes rather than promote hiring decisions.

## 3 Psychological and systemic barriers to employment

### 3.1 Psychological frameworks underpinning employer decision-making

Employer decision-making in response to criminal record disclosure can be understood through three psychological frameworks: stigma theory, dual-process models of judgment, and the affect heuristic. Stigma theory conceptualises criminal convictions as socially salient labels, with the criminal record functioning as a “master status” that overrides individuating information such as skills, offence age, or evidence of rehabilitation (Goffman, 1963; Link and Phelan, 2001). Dual-process models (Kahneman, 2011; Evans and Stanovich, 2013) suggest that hiring decisions are often guided by fast, intuitive reasoning (System 1) rather than deliberative evaluation (System 2), particularly under uncertainty or perceived risk. Heuristic judgments arising from these intuitive processes lead employers to overweigh emotionally charged offences. The affect heuristic (Slovic et al., 2007; Porter et al., 2023, 2025a) further explains why sexual or violent offences inflate perceived risk, producing systematic overestimation of harm. Together, these processes help explain why disclosure-based policies often fail to produce proportionate, evidence-led employment decisions.

### 3.2 Stigma, risk perception, and employer decision-making

Psychological research consistently demonstrates that criminal records function as powerful *stigma markers* in employment contexts. Employers tend to rely on heuristic judgments, overestimating risk and underestimating the relevance of contextual information, such as the age of the offence, rehabilitation, or external supervision.

This research is typically examined using correspondence testing, in-person audit studies, and laboratory-based experimental research (Porter et al., 2025b, 2026). These methodologies allow researchers to directly observe employer and participant reactions to criminal records. Collectively, they demonstrate employment barriers through both experimental designs (Lewis et al., 2025; Porter et al., 2023, 2025a) and field-based research (Rovira, 2024).

In an extensive review of these empirical methodologies, Porter et al. (2026) argue that the substantial variation in research design makes it difficult to synthesize a comprehensive overview of the barriers faced by justice-involved individuals. Consequently, they suggest that targeted research is essential for the development and implementation of effective employment interventions. Using the experimental decision-making research as an example, we will highlight key issues with employability for those with criminal records.

In this body of research, participants are tasked with selecting one of three candidates they wish to hire and are asked to provide justification for their decision. They are also asked to rate how trustworthy, valuable to the company (or business), and how suitable for the role the candidate will be on a 7-point Likert-type scale. It is only after participants have selected a candidate, and explained their rationale, that they are then informed that the candidate has a prior criminal record (or not). Participants then rate the candidate for trustworthiness, suitability, and value, decide whether to hire (or reject) the candidate, and explain their decision-making. In most experiments, ratings post-reveal of a criminal conviction drops drastically, and rejection rates become substantially high. This is especially true for those with sexual offence convictions.

In summary, public and employer perceptions of who is “*hireable*” are strongly influenced by offence type, with sexual and violent offences eliciting particularly high levels of rejection. Importantly, these perceptions often persist even when candidates meet all role requirements, suggesting that exclusion is driven less by job-related risk and more by moral judgment and fear of reputational harm (Lewis et al., 2025; Porter et al., 2025b; Porter et al., 2023, 2025a).

Collectively, these findings highlight that disclosure alone does not guarantee fair assessment and may instead trigger bias if not carefully structured.

## 4 Policy landscape and current initiatives

### 4.1 Legislation and ineligible checks

The number of individuals in the UK holding a criminal record continues to rise, posing ongoing challenges for employment and reintegration. The Rehabilitation of Offenders Act 1974 allows certain convictions to become “spent,” meaning they are generally not required to be disclosed. However, higher-level criminal record checks—including standard or enhanced DBS checks—can still reveal spent convictions, restricting access to employment.

Additionally, ineligible checks are rising. An ‘ineligible check’ occurs when an employer requests a criminal record check at a higher level than is legally permitted for the role, such as an enhanced check where only a standard check is appropriate, or a standard/enhanced check where only a basic check is needed. For instance, a retail worker applying for a cashier position might face an enhanced DBS check that reveals spent convictions, even though the role only requires a basic check. Unlock (2020) found that these ineligible checks are widespread and problematic, leading to unnecessary disclosure of spent convictions and disadvantaging thousands of individuals each year. Such practices not only undermine reintegration and fair access to work but also create legal risks for employers and erode trust in criminal record systems. Addressing ineligible checks is therefore critical for improving employment outcomes, promoting social inclusion, and ensuring the effectiveness of reintegration policies.

### 4.2 Ban the box and deferred disclosure

Current campaigns offer only vague recommendations. For instance, Ban the Box calls on UK employers to give people with criminal records a fair chance to compete for jobs by removing the tick box from application forms, and instead asking about criminal convictions later in the recruitment process. They do not provide recommendations on how employers should be provided with this criminal record information. Porter et al. (2023, 2025a) consistently demonstrate that a DBS check after a candidate is selected negatively affects employability decisions.

While the Ban the Box initiative has demonstrated some positive outcomes, evidence suggests that its effects are uneven and context-dependent. For individuals convicted of highly stigmatised offences, delayed disclosure may simply postpone rejection rather than prevent it. In some cases, it may even increase harm by fostering false expectations or intensifying employer backlash at later stages. Research indicates that when disclosure eventually occurs without structured support, rejection rates remain high, particularly for those with violent (Porter et al., 2025b) or sexual offence convictions (Lewis et al., 2025; Porter et al., 2023, 2025a).

It is also important to recognise heterogeneity within offence categories. Not all offences carry the same risk profile, and time elapsed, context, and evidence of rehabilitation can meaningfully influence employability. Tailored approaches that integrate offence-specific risk assessment with structured support can help balance public protection with opportunities for reintegration, ensuring that interventions are proportionate and effective.

### 4.3 The absence of standardised employer guidance

UK employers currently operate within a fragmented policy environment. Disclosure decisions are shaped by organisational culture, sector-specific anxieties, and inconsistent legal interpretation. This results in wide variability in practice, with some employers conducting nuanced, role-specific assessments and others adopting blanket exclusion policies.

Lewis et al. (2025) demonstrate that external signals—such as a probation reference—can modestly reduce rejection rates, suggesting that employers respond positively to *structured reassurance*. However, such practices are not standardised or widely implemented.

### 4.4 Unintended consequences of well-intentioned policies

A recurring theme in the empirical literature is the divergence between policy intention and empirical outcome. Initiatives designed to promote inclusion may inadvertently reinforce stigma if they fail to account for psychological processes underlying employer judgment.

Policies are also often rolled out without rigorous evaluation, particularly for highly stigmatised groups (Porter and Merdian, 2025). This is problematic, as sexual offence convictions are associated with extreme moral condemnation, categorical thinking, and resistance to counter-stereotypical information. Generic employability interventions may therefore be ineffective—or even counterproductive—when applied uniformly across offence types. This is concerning, given that poorly targeted interventions can fail to improve employment outcomes. Moreover, they also entrench social exclusion, reduce trust in rehabilitation programmes, and waste resources that could be more effectively directed toward evidence-based, offence-specific support.

## 5 Policy recommendations

Based on the evidence reviewed, practitioner insights, and policy guidance, we propose the following recommendations. Where empirical evidence is limited—particularly for highly stigmatised offence groups—recommendations are framed as theoretically promising and warrant pilot evaluation.

1. Introduce statutory post-offer disclosure interviews. Require criminal record disclosure to occur after a conditional job offer, accompanied by a structured, standardised discussion focused on relevance, risk management, and rehabilitation. This approach allows candidates to anticipate the disclosure interview and reduces the likelihood of outright rejection. Introducing statutory post-offer disclosure interviews is a theoretically promising intervention that evidence suggests could reduce early-stage exclusion; however, empirical evaluation for highly stigmatised offence groups remains limited.
2. Standardise employer guidance on criminal record use. Develop national, sector-specific frameworks that clarify lawful disclosure, assessment criteria, and best practices, thereby reducing uncertainty, ineligible checks, and defensive exclusion.
3. Standardise disclosure guidelines for those with a criminal record. Navigating the employment market post-release or following a criminal record is challenging. There are several charities and organisations that offer guidance on how and when to disclose a conviction, which often contradict one another.
4. Create specialist guidance for highly stigmatised offences. Recognise that sexual and serious violent offences require tailored approaches, informed by empirical research on stigma, risk perception, and disclosure effects.
5. Establish an employer accreditation scheme. Establish an employer accreditation scheme, a theoretically promising approach that may encourage transparent, inclusive hiring practices, pending empirical evaluation. Incentivise fair-chance hiring through accreditation schemes for organisations that demonstrate evidence-based, transparent, and accountable recruitment practices. Such accreditation would ensure that participating organisations meet clear standards and are held accountable for their practices.
  Initiatives such as Ban the Box are well-intentioned and may reduce formal exclusion at early hiring stages, but in the absence of complementary mechanisms—such as enforceable accreditation—they do little to counter entrenched stigma. In practice, the policy can function more as a reputational signal for employers than a substantive change in hiring behaviour, with discretionary decision-making simply displaced to later stages.
6. Mandate empirical evaluation of employability initiatives. All employability initiatives should undergo pilot testing and rigorous outcome evaluation—particularly for high-risk or highly stigmatised groups—before national implementation. Evaluation should include both raw numbers and percentages when reporting employment outcomes, as reliance on percentages alone can misrepresent the scale of impact and obscure persistent barriers (Porter et al., 2025c). By combining pilot data with transparent reporting, policymakers and funders can accurately assess effectiveness, identify unintended consequences, and allocate resources to programmes that demonstrably improve employment outcomes for individuals with a criminal record.

Some interventions have empirical support in improving employment outcomes for justice-involved individuals (e.g., Ban the Box for low-stigma offences), whereas other proposals are theoretically motivated and require pilot testing or evaluation (e.g., post-offer disclosure interviews, accreditation schemes).

## 6 Conclusion

Exclusion from employment following a criminal conviction is not an inevitable consequence of risk management but a product of policy design, psychological bias, and insufficient evidence use. This brief demonstrates that initiatives grounded in good intentions may fail—or cause harm—when they do not align with empirical findings on stigma and decision-making. By adopting structured disclosure practices, improving statistical communication, and tailoring interventions for highly stigmatised groups, UK employment policy can better balance public protection with rehabilitation. A coordinated, evidence-led approach is essential if employment is to fulfil its role as a pathway to desistance, dignity, and social reintegration.

## References

[ref1] Devins D. Foley B. (2011). Criminal Records and Barriers to Employment. London: UK Commission for Employment and Skills.

[ref2] Evans J. S. B. T. Stanovich K. E. (2013). Dual-process theories of higher cognition: advancing the debate. Perspect. Psychol. Sci. 8, 223–241. doi: 10.1177/1745691612460685, 26172965

[ref3] Goffman E. (1963). Stigma: Notes on the Management of Spoiled Identity. Englewood Cliffs, NJ: Prentice-Hall.

[ref4] Haslewood-Pócsik I. Brown S. Spencer J. (2008). A not so well-lit path: employers’ perspectives on employing ex-offenders. Howard J. Crim. Justice 47, 18–30. doi: 10.1111/j.1468-2311.2008.00505.x

[ref6] Kahneman D. (2011). Thinking, Fast and Slow. New York: Farrar, Straus and Giroux.

[ref1101] Lewis M. Gavin P. Porter C. N. (2025). Employability Perceptions: does a reference from probation reduce rejection rates? Int. J. Law Crime Justice. 83:100785. doi: 10.1016/j.ijlcj.2025.100785

[ref7] Link B. G. Phelan J. C. (2001). Conceptualizing stigma. Annu. Rev. Sociol. 27, 363–385. doi: 10.1146/annurev.soc.27.1.363

[ref8] McGuire J. (1999). What Works: Reducing Reoffending – Guidelines from Research and Practice, vol. 7 Chichester: Wiley.

[ref9] McGuire J. (2013). ““What works” to reduce re-offending: 18 years on,” in What Works in Offender Rehabilitation: An Evidence-based Approach to Assessment and Treatment, eds. Craig L. A. Dixon L. Gannon T. A. (Chichester: Wiley Blackwell), 20–49.

[ref10] Ministry of Justice (2018). Employing Prisoners and ex-Offenders. London: Ministry of Justice.

[ref11] Ministry of Justice (2023). Employment on release: Statistical Release to March 2023. London: Ministry of Justice.

[ref12] Porter C. N. Finn P. Zloteanu M. (2025c). Misleading percentages: the impact of statistical reporting on the offender employment post-release data. Polit. Stud. Rev. doi: 10.1177/14789299251348864

[ref13] Porter C. N. Gavin P. Merdian H. Zloteanu M. (2025b). Exploring social perceptions of hiring individuals convicted of sexual offences in the United Kingdom: a methodological review. J. Crim. Psychol. 15, 495–508. doi: 10.1108/JCP-10-2024-0101

[ref14] Porter C. N. Gavin P. Zloteanu M. (2026). Employer attitudes towards hiring people with a criminal record: a review of empirical methodologies. Int. J. Law Crime Justice 85:100846. doi: 10.1016/j.ijlcj.2026.100846

[ref15] Porter C. N. Haggar L. Harvey A. C. (2023). Sexual offending and barriers to employability: public perceptions of who to hire. Curr. Psychol. 42, 28799–28811. doi: 10.1007/s12144-022-03841-1

[ref16] Porter C. N. Haggar L. Harvey A. C. Gavin P. (2025a). Sexual misconduct and evaluation of candidate appropriateness for employment within a non-public-facing role. Int. J. Law Crime Justice 80:100721. doi: 10.1016/j.ijlcj.2024.100721

[ref17] Porter C. Merdian H. (2025). “Employability barriers for those with a sexual conviction,” in Developing and Implementing a public Health and Criminological Approach to Tackling sexual abuse: Prevention, Treatment and Integration, eds. McCartan K. Eason A. Senker S. Addis N. Porter C. (Cham: Springer Nature Switzerland AG).

[ref18] Rovira M. (2024). Invisible stripes? A field experiment on the disclosure of a criminal record in the British labour market and the potential effects of introducing ban-the-box policies. Br. J. Criminol. 64, 827–845. doi: 10.1093/bjc/azad063

[ref19] Slovic P. Finucane M. Peters E. MacGregor D. G. (2007). The affect heuristic. Eur. J. Oper. Res. 177, 1333–1352. doi: 10.1016/j.ejor.2005.04.006

[ref20] The Oswin Project (2024). 1 in 4 Working-age People in the UK have some form of criminal Record. Northumberland, UK. Available online at: https://oswinproject.org.uk/1-in-4-working-age-people-in-the-uk-have-some-form-of-criminal-record/ (Accessed January 8, 2026).

[ref21] Unlock (2020). Checked out? Ineligible criminal Record Checks and how to Prevent them. London: Unlock. Available online at: https://unlock.org.uk/wp-content/uploads/2020/07/Checked-out_FINAL.pdf (Accessed January 8, 2026).

[ref22] Unlock (2024). New Figures confirm more than 12.5 million People in the UK have a criminal Record. Available online at: https://unlock.org.uk/new-figures-confirm-more-than-12-5-million-people-in-the-uk-have-a-criminal-record/ (Accessed January 8, 2026).

